# Extraction of Rice Phenological Differences under Heavy Metal Stress Using EVI Time-Series from HJ-1A/B Data

**DOI:** 10.3390/s17061243

**Published:** 2017-05-30

**Authors:** Shuyuan Liu, Xiangnan Liu, Meiling Liu, Ling Wu, Chao Ding, Zhi Huang

**Affiliations:** School of Information Engineering, China University of Geosciences, Beijing 100083, China; liushuyuan.456@163.com (S.L.); liumeiling427@126.com (M.L.); wl_19830807@sohu.com (L.W.); dc@cugb.edu.cn (C.D.); zhihuang@cugb.edu.cn (Z.H.)

**Keywords:** phenology, heavy metals stress, rice, HJ-1A/B CCD, EVI

## Abstract

An effective method to monitor heavy metal stress in crops is of critical importance to assure agricultural production and food security. Phenology, as a sensitive indicator of environmental change, can respond to heavy metal stress in crops and remote sensing is an effective method to detect plant phenological changes. This study focused on identifying the rice phenological differences under varied heavy metal stress using EVI (enhanced vegetation index) time-series, which was obtained from HJ-1A/B CCD images and fitted with asymmetric Gaussian model functions. We extracted three phenological periods using first derivative analysis: the tillering period, heading period, and maturation period; and constructed two kinds of metrics with phenological characteristics: date-intervals and time-integrated EVI, to explore the rice phenological differences under mild and severe stress levels. Results indicated that under severe stress the values of the metrics for presenting rice phenological differences in the experimental areas of heavy metal stress were smaller than the ones under mild stress. This finding represents a new method for monitoring heavy metal contamination through rice phenology.

## 1. Introduction

With rapid development of the industry, rice paddies in some areas have been subjected to heavy metal pollution. According to the related statistics, more than 12 million tons of grains are contaminated in China every year [1,2,3]. Heavy metal pollution in croplands has the characteristics of concealment, permanence, irreversibility and toxicity [4]. The issue of food security affecting agricultural production and people’s livelihoods has received much attention [3,4,5,6], and underlines the need to develop effective methods to monitor heavy metal stress in agricultural crop growth.

At present, most research on heavy metals in rice are carried out from the aspects of chlorophyll, leaf area index, cell structure (using a handheld radiometer for extracting spectral or crop parameter information in the field) [7,8,9], or by adopting empirical or semi-empirical models which were established based on the relationships between sensitive spectral characteristics and the heavy metal concentrations, or physiological parameters of crops for the dynamic monitoring of heavy metal stress [10,11,12,13]. These studies are predominantly based on the growth parameters above ground and root weight under heavy metal stress, and these methods are not easy to directly implement. Whereas phenology, which refers to seasonal biological life stages driven by environmental factors, is considered to be a sensitive and accurate indicator of environmental change [14,15,16], the way heavy metals affect rice crop growth is by interfering with the physiological activities of plants, such as photosynthesis, gaseous exchange, and nutrient absorption, to cause reductions in plant growth and dry matter accumulation [17,18]. Thus, these effects may lead to changes on rice phenology. Research has also shown the effects of heavy metals on rice phenology in the laboratory environment, for example, when rice is under cadmium (Cd) stress, plants are thin, leaves are short, the tips of the leaves in the middle and lower part are withered and yellow, and the heading date is delayed for three to four days [19]. Higher concentrations of Cd (500 ppm) decrease secondary growth [20]; furthermore, when rice is harmed by arsenic (As), the amount of tillering will reduce and the heading period will be slightly delayed [19]. The above-mentioned results were based on the laboratory environment and tested the impacts of some heavy metal on rice growth. However, in the natural environment, it is often associated with a variety of heavy metals that affect the crop growth. As rice phenological information is of great importance for agricultural monitoring [21], it is necessary to identify the phenological differences under varied heavy metals stress for monitoring rice phenology. 

Compared with methods based on traditional ground remote sensing and model simulation, extracting the rice phenological information through satellite remote sensing to monitor heavy metal stress is more convenient and direct. Knowledge of crop calendars and phenology is often a key element in vegetation interpretation. Since the 1970s, the potential of multi-temporal satellite observations to provide information on the phenological development of natural vegetation and crops have been recognized by many researchers [22,23,24]. Previous studies on phenology have been mainly based on time-series analysis of remote sensing data to provide important information for detecting crop phenological periods [25,26,27]. The inversion of vegetation indices (VI), such as the normalized difference vegetation index (NDVI) and enhanced vegetation index (EVI) to extract crop phenology is the most common method [28]. VIs based on spectral transformations of two or more bands are designed to improve the contribution of vegetation characteristics and allow dependable spatial-temporal inter-comparisons in terrestrial photosynthetic activity and canopy structural variations. In addition, the information on the timing and progression of paddy rice development may help researchers to infer the condition of plants and their environment (e.g., soil factors) [22]. Meanwhile, heavy metals as a soil factor effects crop growth as they are taken up by the roots from the soil and transported to the aboveground parts of plants [10]. Hence, the investigation of the relationship between phenology and heavy metal stress in rice is helpful in understanding the mechanisms of plants responding to the concentration of heavy metals in the soil, and allow more precise predictions about the effects of future concentration of heavy metals in the soil on rice phenology.

The objective of this study was to explore the potential of evaluating heavy metal stress based on rice phenology extracted by remote sensing technology. In order to verify the effectiveness of using phenological characteristics to monitor heavy metal stress in rice, we compared and analyzed the phenological differences under mild and severe heavy metal stress by two kinds of metrics with phenological characteristics for proving that the specific phenological characteristics are effective indicators for monitoring heavy metal stress in rice. The findings are expected to assist in monitoring the heavy metal contamination of rice and measure the degree of regional heavy metal pollution from the perspective of phenology.

## 2. Study Area and Materials

### 2.1. Study Area

**Figure 1:**
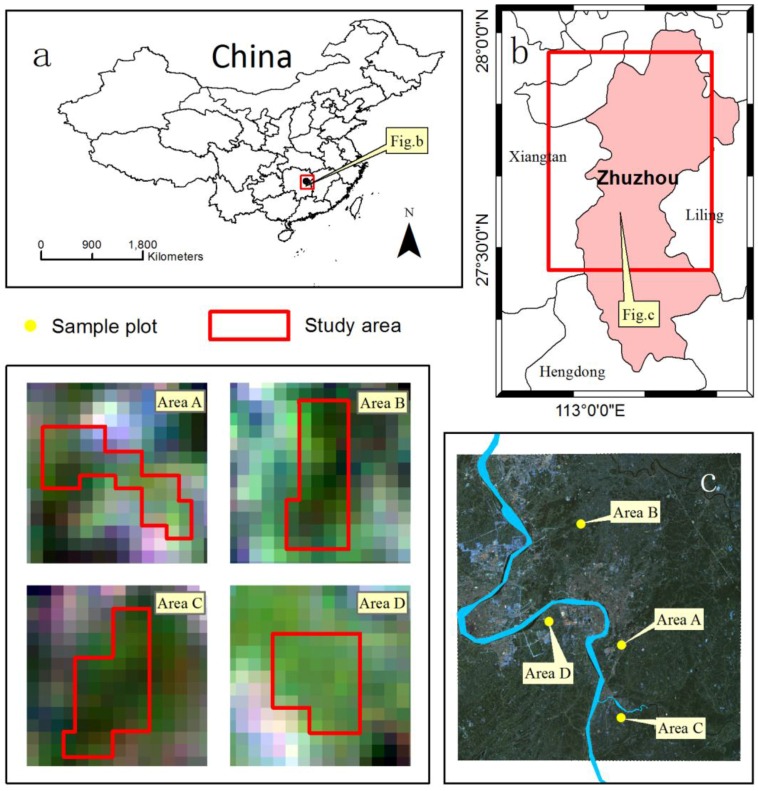
The experiment areas A, B, C, and D in Zhuzhou, Hunan Province in China and the spatial distribution of sample points.

The city of Zhuzhou, in the Hunan Province, China, is an important commodity grain producing area and, simultaneously, is an old industrial base. From the industrial activity, the Xiangjiang River and its tributaries were contaminated. The main heavy metals in this area are cadmium (Cd), plumbum (Pb) and arsenic (As). Four rice fields adjacent to the Xiangjiang watershed in Zhuzhou were selected as experimental fields (Figure 1), and each field site was around 1.28 km×1.28 km in size. And in this area, the main type of rice grown is hybrid rice Boyou 9083. Furthermore, the main soil type is red soil with sufficient organic matter content (2–3%), and the dominant climate is subtropical monsoon with a mean annual temperature of approximately 16–18 °C. The annual average precipitation is 1257 mm and the total number of sunshine hours during the rice growing season is 969.6 h. Heavy metal stress levels were determined as mild or severe according to the analysis of soil sample (Table 1) and varied between the four experimental regions (labeled A, B, C, and D). The samples of soil were collected in five sample plots from each of the four experiment areas at three important rice phenological stages (initial tillering stage (mid-June), the middle tillering stage (mid-July), and the maturation stage (late August)), and the corresponding statistical information are shown in Table 2. The collected soil samples sited at rooting zone, and according to the *Environmental Quality Standard for Soils* in China [29], the depth of collected soil samples was 0–20 cm [30]. In these rice paddies, the intensive planting pattern made the impact of the characteristic spatial variability from the content of soil nutrients and soil texture smaller. Furthermore, the four rice experimental fields were cultivated and irrigated adequately to avoid other unnecessary stress caused by other environmental factors, such as the nutrient deficiency, weeds, pests, etc. With the exception of the above factors effecting similarly on rice crops, the four experimental areas were only different in human management and soil heavy metal stress.

### 2.2. Data Preparation

In this study, 16 CCD images from the small sun-synchronous satellites, HJ-1A/B, which are used for monitoring and forecasting environmental and natural disasters, were adopted as the remote sensing data covering four experimentation areas in the entire growing season of rice paddies (dated from 13 June to 29 September 2013). The HJ-1A/B satellite can cover a ground swath 700 km wide, and two CCD sensors were loaded on satellites which formed a constellation to constitute an observation network that covered China and its surrounding areas on a two-day-repeat cycle. The CCD sensors captured ground features with a 30 m pixel resolution at a nadir angle in the visible bands. These CCD images consisted of the following four channels: band 1: 0.43–0.52 μm (blue waveband), band 2: 0.52–0.6 μm (green waveband), band 3: 0.63–0.69 μm (red waveband), and band 4: 0.76–0.90 μm (near-infrared waveband). Wang et al. [31] integrated HJ-1A/B CCD and Landsat-8 OLI vegetation indices time-series images to estimate phenological information in rice that demonstrated the good quality of HJ-1 A/B CCD images; Pan et al. [32] used CCD images from HJ-1A/B to map crop phenology. Given the capabilities of HJ-1A/B CCD images, it is suitable for extracting crop phenological information. Due to the monsoon climate, during the growth phase of the paddy rice, only 16 cloud-free CCD images could be obtained, and the number and distribution of these 16 images are sufficient to meet the experimental requirements. Thus, in this paper, we took advantage of all the above-mentioned to construct a vegetable index time-series dataset, making it possible to extract rice phenology.

The time is expressed as the day of the year (DOY) in this paper. Among the 16 images (six for the tillering period; four for the heading period; six for the maturation period), there were two images in June (DOY168 and DOY181), four images in July (DOY189, DOY193, DOY206, and DOY210), five images in August (DOY219, DOY221, DOY223, DOY225, and DOY241), and five images in September (DOY252, DOY258, DOY262, DOY264, and DOY272). All of the 30 m multispectral CCD images were processed prior to use, and the specific process can be summarized as: (1) radiometric-calibration was performed for each band, and the four bands of each scenes were packed; (2) the CCD images were clipped to obtain a subset (55,650 m×59,220 m) where the four experimental areas are located; (3) the data format was converted to BIL and performing atmospheric-correction by using the FLAASH module embedded in the ENVI 5.2 software (Exelis VIS, White Plains, NY, USA); (4) the projection of all images was made uniform; (5) geometric correction for all images based on a specific image was performed by using automatic image registration embedded in ENVI 5.2 (Exelis VIS, White Plains, NY, USA); (6) the EVI for each image was calculated; and (7) the EVI images were layer stacked to construct time-series stacks for 2013.

## 3. Methods

### 3.1. Preprocessing of Multi-Temporal Satellite Data

#### 3.1.1. Calculation of Vegetation Index

The extraction of vegetation phenological information by remote sensing are mostly based on the vegetation indices time-series curves, that is, the vegetation indices of time-series using remote sensing images are analyzed by smoothing and parameter analysis of the vegetation indices to extract phenological information while reflecting seasonal characteristics of vegetation community for temporal-spatial analysis [33,34,35,36]. Therefore, the choice of vegetation indices is very important and should be highly sensitive to reflect changes in the ground environment. 

NDVI is the most widely used vegetation index for monitoring vegetation phenology. EVI, the enhanced vegetation index based on NDVI, is applied increasing widely. To a certain extent, the two VIs can reflect the comprehensive situation of the land cover types from pixels in corresponding regions. The periodic increase and decrease of the VIs value of the region is a typical performance of the vegetation growth cycle, which can be used to determine the vegetation growth and overall situation. Additionally, the differences of VIs among different regions can reflect the vegetation, soil type, precipitation, topography and land use, and so on [37]. Thus, the inversion of vegetation indices is an effective method to analyze the changes of regional vegetation, which is to observe the changes of the VIs curves: vegetation grows, VIs increase; vegetation dies, VIs decrease. However, compared with the NDVI, EVI uses the “anti-atmospheric vegetation index” and “anti-soil vegetation index” to overcome several disadvantages, such as the influence of soil background; NDVI is easily saturated in high vegetation coverage, and is easily affected by soil and vegetation in low vegetation coverage; the atmospheric attenuation is not completely removed, and so on [38]. In addition, EVI can be linear dependence with LAI (the leaf area index), and has a higher sensitivity compared with that of NDVI in high biomass areas [37], so it can be used to detect the process of the high biomass of rice paddy growth adequately with a greater dynamic range [34]. Thus, we used EVI instead of NDVI for rice phenology detection. The EVI formula is given as follows:

(1) $$\mathrm{EVI}=2.5\times \frac{\rho_{NIR}-\rho_{RED}}{\rho_{NIR}+C_{1}\times \rho_{RED}-C_{2}\times \rho_{BLUE}+L}$$

where $\rho_{NIR}$, $\rho_{RED}$ and $\rho_{BLUE}$ are the reflectance of near-infrared band, red band, and blue band corresponding to the values of Band 4, 3, and 1 in the HJ-1A/B CCD sensors, respectively. L and the coefficients C1 and C2 describe the use of the blue band in correction of the red band for atmospheric aerosol scattering. The coefficients, C1, C2, and L, are empirically determined as 6.0, 7.5, and 1.0, respectively [39].

#### 3.1.2. Reconstruction of EVI Time Series

Due to the presence of cloud, shadow, and other factors, the time-series EVI data still had a lot of noise, so they need to be filtered and reconstructed before application [40]. In the field of phenology monitoring, the TIMESAT program is currently used, and the software has a special webpage to introduce the software related information [41]. The software integrates AG (asymmetric Gaussian model functions), DL (double logistic model functions), and SG (Savitzky-Golay filtering method) filtering algorithms, which can process time-series and image data (two-dimensional space array) simultaneously [42,43].

Many researchers performed the studies about the fidelity performance and the capability of keeping main characteristics on the three algorithms for vegetation phenology detection. Wang, Xin, and Shu [40] used EVI time-series to compare three methods, and thought that the results using AG algorithm based on the envelope of the fitting can be closer to the real value, but the peak reconstruction performance is slightly worse than that using the DL algorithm, especially for shrub and grassland type in China. Hird and McDermid [44] performed an in-depth comparison of AG, DL, SG, and other reconstruction methods to reconstruct NDVI, and found that they all have great filtering ability, but AG and DL have better reconstruction performance overall. Cao, Wang, and Den [45] considered that the fidelity performance of AG is the best, followed by DL, and SG is relatively poor for high-quality MODIS NDVI data reconstruction. However, the phenological differences under varied heavy metal stress are small, at approximately 1–5 days [19]. Thus, in order to extract the slight differences under varied heavy metal stress on rice phenology, the asymmetric Gaussian model function (AG), which has better fidelity performance and reconstruction performance overall was selected to de-noise and fit the EVI time-series in this study. The method is based on the nonlinear least squares fits of asymmetric Gaussian model function, which can define key seasonality parameters, such as the number of growing seasons, the beginning and end of seasons, and the rates of growth and decline [42]. In the TIMESAT software, the EVI time-series of the experimental areas extracted the information of pixels by pixel to check for anomalous events, missing data, and noisy time-series.

### 3.2. Detection of Rice Phenology

After the original data were smoothed, the temporal profiles of EVI were obtained. Next, according to the characteristics of EVI time-series curves, the specific phenological periods were extracted. The rice growth period was from transplanting time to harvest time. Remote sensing in rice using optical satellite sensors exploited the phenological characteristics of the rice life-cycle. About one to two weeks after transplanting, the rice turned into the tillering period. In the tillering period, due to the development of rice root and leaf systems, EVI rapidly increased until the heading period when the vegetative development of rice reached its maximum, and rice growth becomes a descending stage where EVI decreases gradually, due to the nutrients transferred into the seeds. During the maturation period, due to the loss of most of the chlorophyll, the leaves rapidly withered, and the vegetation index was minimized in the harvest period [35]. As mentioned above, we used first derivative (FD) analysis to identify the dates of rice key phenology transitions. First derivative analysis is also called the slope method, and the specific crop growth period is determined by the magnitude of vegetation growth rate in the temporal profiles of EVI [46,47]. The FD formula is given as follows:

(2) $${\mathrm{FD}}_{\mathrm{EVI}}=\frac{\left({\mathrm{EVI}}_{\left(i+1\right)}-{\mathrm{EVI}}_{i}\right)}{\Delta_{\mathrm{DOY}}}$$

where ${\mathrm{FD}}_{\mathrm{EVI}}$ is the FD value of EVI between the i th and i + 1 th DOY; the value of ${\mathrm{EVI}}_{i}$ is at the i th DOY; the value of${\;\mathrm{EVI}}_{\left(i+1\right)}$ is at the i + 1 th DOY; and $\Delta_{\mathrm{DOY}}$ equals 1 as the interval. 

Based on the analysis method of first derivative, we selected the appropriate indicators to identify the rice growth stage: (1) due to a high level of LAI and biomass in the heading period (mid-August), this period will have the maximum EVI corresponding to the value of FD (FD_0_) [36,47]; (2) in the tillering period, rice plants grow rapidly, and the highest speed might be at the active tillering stage (mid-July) [27]. Thus, the maximum value of FD (FD_max_) was used to estimate the active tillering period; and (3) similarly, at the end of the maturation stage, EVI declines rapidly due to leaf senescence [27]. Hence, we selected the minimum value of FD (FD_min_) to detect the maturity period (mid-September). According to the characteristics of rice growth stages described above, the identification of rice growth and development period can be detected through the vegetation index of remote sensing image time-series.

### 3.3. Establishment of Rice Phenological Metrics

**Figure 2:**
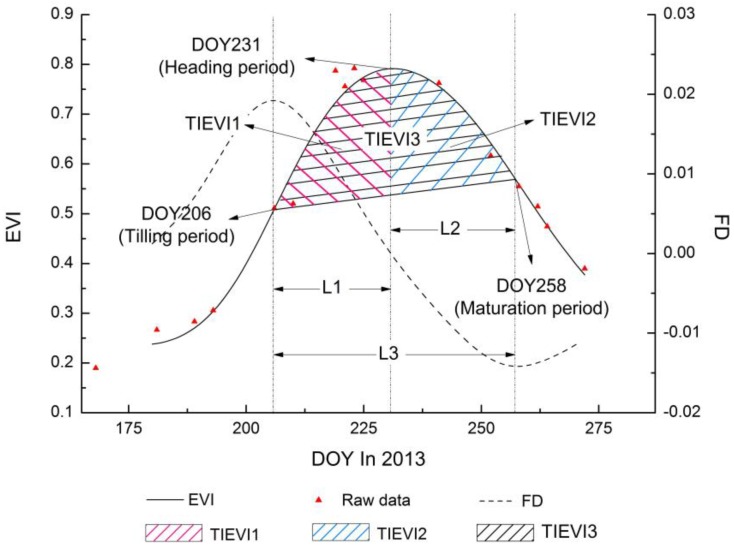
The lengths of time (L1, L2, and L3) and the integral areas (TIEVI1, TIEVI 2, and TIEVI 3) under the EVI time-series curve between three phenology periods (tilling period, heading period, and maturation period) were marked on the temporal profiles, respectively.

As the four experimental areas were from different places, there were differences in the specific transplanting period (mostly in mid-June) and the definition of phenological periods by traditional methods, therefore, we could not identify the delay and advance for the specific phenological period. To detect the impact of heavy metals in soil on rice phenology, we selected the date-intervals, which meant the lengths of time between different phenological phases and eliminated the impact of different planting time, as the heavy metal stress metrics of rice phenology to extract the same differences under varied heavy metal stress levels. The specific metrics are calculated as follows (Figure 2):

(3) $$L1={\mathrm{DOY}}_{\text{Heading period}}-{\mathrm{DOY}}_{\text{Tilling period}}$$

(4) $$L2={\mathrm{DOY}}_{\text{Maturation period}}-{\mathrm{DOY}}_{\text{Heading period}}$$

(5) $$L3={\mathrm{DOY}}_{\text{Maturation period}}-{\mathrm{DOY}}_{\text{Tilling period}}$$

where ${\mathrm{DOY}}_{\text{Tillering period}}$, ${\mathrm{DOY}}_{\text{Heading period}}$, and ${\mathrm{DOY}}_{\text{Maturation period}}$ are the day of year of three phenological periods in 2013. L1, L2, and L3 refer to the lengths of time between three phenology periods (tilling period, heading period, and maturation period), respectively. 

To further analyze the effect of heavy metal concentration on rice phenology, the differences between L1 and L2 under same heavy metal stress were taken as a measure, that is, the variation tendency of two adjacent phenological intervals (between the tillering period and heading period and between the heading period and maturity period) under different heavy metal stresses was analyzed, and the formula is as follows:

(6) $$\Delta_{i}=L1-L2$$

where i represents the four experimental areas: Area A, Area B, Area C, and Area D. $\Delta_{i}$ is the difference between L1 and L2.

The dates of the tillering, heading, and maturation periods can be extracted by remote sensing data using the phenology monitoring algorithms, as the EVI time-series reflect changes in the morphological and physiological condition of the rice during the rice growth cycle [28]. The time-integrated NDVI means the integral areas under NDVI time-series curves can describe the size of the rice growing season [22,48,49]. Furthermore, Reed et al. [22] verified that the time-integrated NDVI metric of phenological interpretation is net primary production, had strong coincidence with predicted phenological characteristics. Therefore, we assumed that the time-integrated EVI metric also described the size of the rice growing season between different phenological periods and could be used to reveal net primary production. This type of metric may not necessarily correspond directly to conventional, ground-based phenological events, but provides indicators for ecosystem dynamics. The specific metrics are calculated as follow (Figure 2):

(7) $$\mathrm{TIEVI}1=\int_{DOY_{Tilling\;period}}^{DOY_{Heading\;period}}F{\left(x\right)}_{EVI}dx$$

(8) $$\mathrm{TIEVI}2=\int_{DOY_{Heading\;period}}^{DOY_{Maturation\;period}}F{\left(x\right)}_{EVI}dx$$

(9) $$\mathrm{TIEVI}3=\int_{DOY_{Tilling\;period}}^{DOY_{Maturation\;period}}F{\left(x\right)}_{EVI}dx$$

where $F{\left(x\right)}_{EVI}$ represents functions of the EVI time-series curves. TIEVI1, TIEVI 2, and TIEVI 3 equal the integral areas under the EVI time-series curves between three phenological periods (tilling period, heading period, and maturation period), respectively.

## 4. Results

### 4.1. Extraction of Rice Phenology

**Figure 3:**
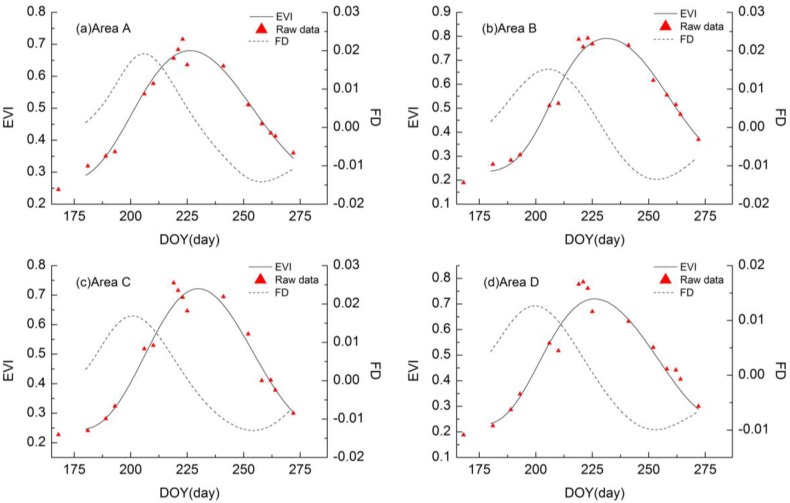
The raw data on EVI from remote sensing image, the fitting curves of EVI time-series by AG function, and their corresponding FD curves for four pixels from the four experiment areas (Area A, Area B, Area C, and Area D), respectively.

In this study, approximately 50 pixels from CCD images were extracted in each of the four experimental areas to fit the time-series of the EVI and extract phenological periods by pixel, before averaging the pixel information in the same experimental area. Figure 3 shows the raw data on EVI from remote sensing images, the fitting curves of EVI time-series by the AG function, and their corresponding FD curves for four pixels from the four experiment areas. As shown in Figure 3, all the smoothed curves of the EVI time-series presented a similar trend, as did the FD curves. The fitting curves of the EVI were much closer to the raw data values. At the beginning of the rice growth stage, the values of the EVI were comparatively low; however, the EVI between DOY180 and DOY190 increased rapidly. Simultaneously, FD also increased rapidly and corresponded to the time when the rice crops began to produce tillers and the green biomass increased rapidly. When FD obtained peaks at about DOY205, the EVI had the maximum increasing rate when the tillering period was most active. At approximately DOY230, the EVI reached the maximum that marked the rice crops entering into the heading period. After that, the values of the EVI stopped increasing and began to descend, and when FD got the valleys at about DOY257, the EVI had the maximum descending rate when the rice crops reached the maturation stage. 

**Figure 4:**
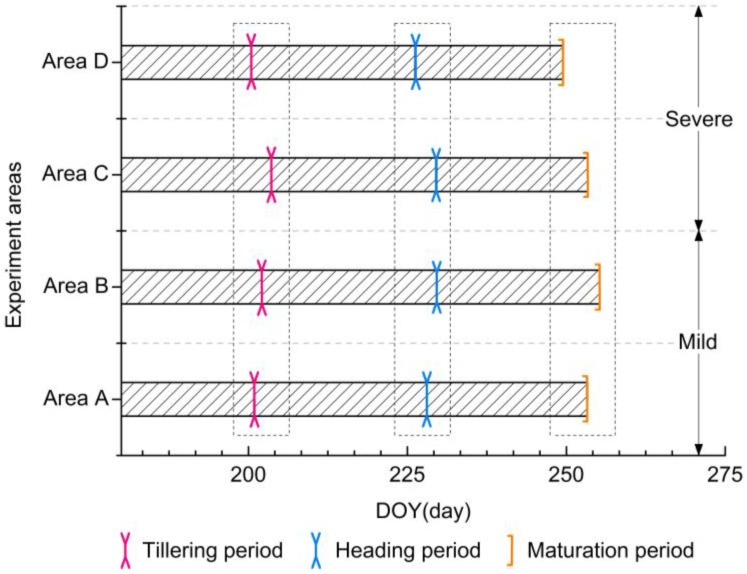
The averages of dates corresponding to the three phenological periods(the tillering period, the heading period, the maturation period) in the four experiment areas (Area A, Area B, Area C, and Area D), respectively.

According to first derivative (FD) analysis (FD_max_, FD_0_, and FD_min_) of smoothed EVI temporal profiles, three phenological periods (the tillering period, the heading period, the maturation period) in the four experiment areas were obtained and averaged, respectively. Figure 4 represents the averaged of dates corresponding to the three phenological periods from the four experimental areas. As seen in Figure 4, the tillering periods in Area A and Area D were at DOY200, approximately, and ca. DOY203 was revealed as the tillering periods in Areas B and C. As for the heading periods, Areas B and C had the closer dates (around at DOY230), and the dates of Areas A and D were at DOY228 and DOY225, respectively. During the maturation periods, the specific dates of the four experimental areas had significant differences: Areas A and C were at DOY253, Area B was at DOY255, and Area D was at DOY249. More detailed information on the standard deviation of dates corresponding to the three phenological periods is provided in Table 3. However, the existence of differences on specific phenological periods between the four experiment areas is partly due to the different transplanting periods in rice from the four experimental areas. In addition, according to the above results, it was difficult to observe the specific effects on rice phenology under heavy metal stress. Thus, according to above-results, some metrics were necessary for detecting the effects of heavy metals stress on rice phenology.

### 4.2. Phenological Differences in Rice under Heavy Metal Stress

#### 4.2.1. Differences in the Intervals of Rice Phenological Periods under Heavy Metal Stress

**Figure 5:**
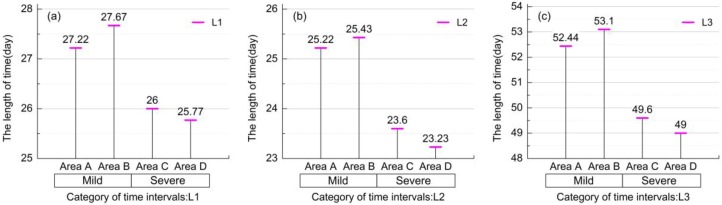
Comparison of the DOY lengths of time between three phenological periods (the tillering period, the heading period, and the maturation period): (**a**) L1 exhibits the time intervals between the tillering period and heading period, (**b**) L2 exhibits the time intervals between the heading period and maturation period, and (**c**) L3 exhibits the time intervals between the tillering period and maturation period, from the four experiment areas (Area A, Area B, Area C, and Area D), respectively.

To analyze the differences on rice phenology under two heavy metal stress levels, the lengths of time between three phenological periods (L1, L2, and L3) were selected as the metrics to present the phenological differences under stress. Figure 5 shows the comparison of the three metrics from the four experimental areas, respectively. Table 4 presents the statistical information on L1, L2, and L3. Overall, the values under mild stress (Areas A and B) were higher than the ones under severe stress (Areas C and D) in various degrees, thus indicating that there was a stress effect on the three phenological periods of rice in Areas C and D. The lengths of time between the tillering period and the heading period under mild stress in Areas A and B were longer approximately 1–2 days than in Areas C and D, and the phenological differences under mild stress level were about half a day, with smaller differences under severe stress (Figure 5a). Furthermore, between the heading and maturation periods, there were two days’ differences under different stress; however, under the same stress levels, there were some minimal differences (Figure 5b). This result indicated that under the same stress, the effects of different concentrations of heavy metals on rice phenology during the tillering period and the heading period were less, and as were the effects during the heading and maturation periods. In addition, there were minimum values in Area D where all values of heavy metals concentration were the maximum (Figure 5a–c). The two metrics, L1 and L2 could exhibit the phenological differences under the two stress levels in a relatively small degree; however, the whole length of phenological periods between the tillering and maturation periods had a large decline where there were about three days under the two stress levels at least, and the longest gap was almost four days between Areas B and D. Thus, it was more obvious to measure the heavy metal stress levels using longer lengths of time between phenological periods. This observation illustrated that there are phenological differences under heavy metal stress levels by extracting phenological information from the EVI time-series of remote sensing data, and at the same time, demonstrated the abilities of the metrics, L1, L2, and L3, to distinguish heavy metal stress levels. Thus, the longer the interval between phenological periods, the greater the differences of metrics.

**Figure 6:**
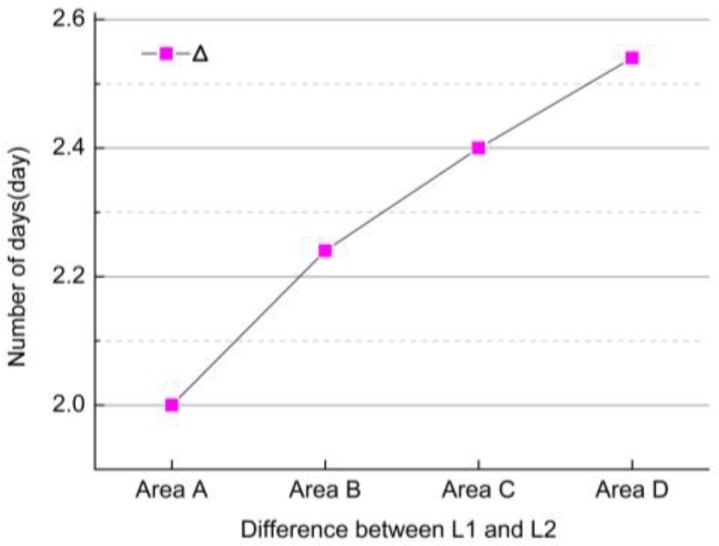
The differences ($\Delta_{i}$) between L1 and L2 varied from the four experiment areas (Area A, Area B, Area C, and Area D), respectively.

Different concentrations of heavy metals had distinct effects on the rice different growth stages. As shown in Table 1, the concentrations of heavy metals gradually increased from Area A to Area D. Figure 6 shows that the differences between L1 and L2 also gradually increased from Area A to Area D. This result revealed the variation tendency of adjacent two phenological intervals (between tillering period and heading period and between heading period and maturity period) with different concentrations of heavy metals. In detail, the lengths of time during the tillering and the heading periods were gradually longer with increased heavy metal concentration of the four experimental areas, but the ones during the heading and maturation periods were gradually shorter.

#### 4.2.2. Differences in the Time-Integrated EVI of Rice Phenology under Heavy Metal Stress

**Figure 7:**
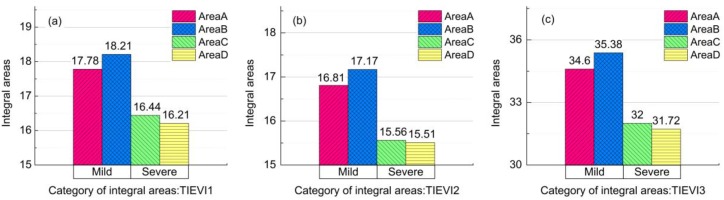
The integral areas of EVI time series during the three phenology periods (the tillering period, the heading period, the maturation period): (**a**) TIEVI1 exhibits the integral areas between the tillering period and heading period, (**b**) TIEVI2 exhibits the integral areas between the heading period and maturation period, and (**c**) TIEVI3 exhibits the integral areas between the tillering period and maturation period, from the four experiment areas (Area A, B, C, and D), respectively.

Different characteristics of rice growth status presented different responses under heavy metal stress. Based on the time-series curves constructed by the EVI values obtained from the remote sensing images, the trend and abundance of curves can reflect the size of rice growth season. Figure 7 shows the values of metrics, TIEVI1, TIEVI2 and TIEVI3, which are the integral areas of the EVI time-series between three phenological periods, came from four experimental areas. As shown in Figure 7a–c, the highest values of the integral areas were in Area B, and the values of TIEVI1, TIEVI2, and TIEVI3 in Area A under mild heavy metal stress were comparatively lower than in Area B, but higher than the two other areas under severe heavy metal stress. Thus, it was revealed that there was a stress effect on rice phenology under the severe stress level. In Areas C and D under the severe heavy metal stress, there were closer values of the integral areas between different phenological periods, and the values were minimum in Area D where the values of heavy metal concentration reached the maximum. This result illustrated that there are the closer effects of different concentrations of heavy metals on rice phenology during the heading and maturation periods under severe stress levels. However, under mild stress level, Areas A and B, showed a relative decline, which revealed that effects of different concentrations of heavy metals on the rice phenology during the tillering and maturation periods under mild stress were varied. On the margin of differences, TIEVI3 had better abilities to present the differences under the two kinds of heavy metal stress levels (Figure 7c). The statistical information of TIEVI 1, TIEVI 2, and TIEVI 3 on standard deviation is shown in Table 5. In this part, we selected other kinds of metrics with phenological features to try to extract the phenological differences under the two kinds of heavy metal stress levels, and considered that the results of comparing integral areas between three phenological periods could be the metrics to refer to the stress levels, especially TIEVI3. 

## 5. Discussion

This study presents a new methodology for monitoring heavy metal stress in crops based on crop phenology with remote sensing technology. The methodology was identified through extracting rice phenological differences under mild and severe heavy metal stress levels by the EVI time-series, which was obtained from HJ-1A/B CCD images and fitted with asymmetric Gaussian model functions (AG). With first derivative (FD) analysis, three phenological periods (the tillering peirod, the heading period, and the maturation period) were detected, and then two kinds of metrics with phenological characteristic: date-intervals and time-integrated EVI were constructed and compared in the four experimental areas. The results indicated that the phenology is an effective indicator for monitoring heavy metal stress in rice.

It is critical that phenological differences under different heavy metals stress are measured. In this study, we selected the EVI time-series, which were composed of CCD images, to define three key phenological periods. CCD images from HJ-1A/B have high temporal-spatial resolution were obtained, but due to the monsoon climate, the cloud-free remote sensing data during the growth phase of the paddy rice was limited. Compared with other remote sensing data commonly used for monitoring vegetation phenology, CCD data represents the value of the day unlike AVHRR and MODIS remote sensing data of usually eight or 16 days of synthetic products, which means any one of the images were compounded by the optimal algorithm between eight days or 16 days. Thus, we can obtain better and closer to the true value of smoothed curves of the EVI time-series that also contributed to detect phenological differences under the two heavy metal stress levels. 

Some studies found that the crop growth under heavy metal stress becomes slower comparing with non-contaminated conditions [50,51,52,53]. However, in our study, what is new is that two kinds of metrics with phenological characteristics were selected for comparison under two heavy metal stress levels. In our results, comparing the lengths of time between different phenological periods of rice under two heavy metal stress levels showed that the values under severe stress were shortened. The results could be explained by the time-integrated EVI as an indicator with phenological characteristics for ecosystem dynamics. Firstly, some studies have shown that the presence of heavy metals, Cd, Pb, and As in soil, can inhibit rice crop growth and the formation of rice grain yield in different ways [53,54]. In addition, the EVI, which represents crop growth status, can show linear dependence with LAI (the leaf area index) [37]. These possibly resulted in the differences on the values of the EVI obtained from remote sensing images at different phenological periods of rice in the four experimental areas that may help to bring about the results. Secondly, in our study, the dates corresponding to the extracted three rice phenological periods in the four experimental areas were relatively close, but there were slight differences. This may be partly due to heavy metal stress; partly due to the different transplanting periods in rice from the four experimental areas. The extracted three rice phenological periods corresponded to a certain moment in the phenological stages, respectively, that we could not identify the delay and advance for the specific phenological period, or even obtain the length of the whole rice growing season. This showed that the metrics for detecting rice phenological differences under varied heavy metal stress are necessary, and the result was related to the meaning of the metrics. Moreover, according to study by Du et al. [53], at the active tillering stage, the inhibition of cadmium on rice tillering is more obvious; after the active tillering stage, the inhibition gradually reduces. We suspected that different degrees of change in different phenological stages also contributed to the results. The results presented in this study suggest that the two kinds of metrics, the length of phenological periods and the integral areas can be considered as useful indicators for monitoring heavy metal stress in rice plants and could be deemed as a label of mild or severe stress levels for the regions in this study: L3 ≤ 49.6 or TIEVI3 ≤ 32 as severe stress, conversely as mild stress or no stress. Beyond the values of stress levels in this study, the changes in rice phenology became unpredictable. In addition, the variations of differences between L1 and L2 with increasing concentrations of heavy metals revealed the variation tendency of adjacent two phenological intervals with different concentrations of heavy metals, and the difference between the four experimental areas was small. This may be because the degree of heavy metal stress influence on the length of time between the tillering period and the heading period is different than that between the heading period and the maturation period [53].

This study provides a new way to monitor heavy metal pollution in agriculture through remote sensing technology. Compared to traditional ground-based methods used to monitor heavy metal stress, remote sensing is a simple and convenient resource. Furthermore, the analysis of phenology presented here concerned the effects of temperature, climate, rainfall, and so on. In this study, the results of the metrics’ analyses clearly indicate that heavy metal stress is also an important factor that affects phenological changes. At present, in the case of serious heavy metal pollution, when investigating the phenological changes of terrestrial ecosystems, it is necessary to consider the impact of heavy metal pollution in heavy metal contaminated areas. 

However, it is necessary to declare that the relationship between the phenological differences and stress metrics found in this study are specific to the selected phenological periods studied (the tillering period, heading period, and maturation period), and the results might differ for other phenological periods monitored, other types of data used, other smoothed functions, other measures of phenological period estimation, and other stress metrics. Thus, when the metrics are applied to other areas or other crops, another comparative analysis should be undertaken. Furthermore, the values of the metrics presented in this study ought to be proved. In conclusion, the generality of this finding in other areas and crops require further research.

Our results are limited by the selected phenological extraction method’s sensitivity and fidelity and the metrics used, but under the severe stress level, the length of time between the tillering period and the maturation period was around 49 days, and the integral areas of the EVI time-series during the tillering and maturation periods was about 32. These were identified via the statistical properties, and a comparative analysis of the metrics is an important indicator of monitoring phenological differences under mild and severe stress levels to fully investigate heavy metal stress levels of rice paddies based on extracting phenological periods from EVI time-series curves by remote sensing data and the fitting method. Meanwhile, it should be pointed out that, in this study, two kinds of metrics were qualitatively analyzed, and the performance of the metrics could be further tested through detailed quantification using measured data and more detailed procedures. The results in this paper could be further verified by collecting field observation data. In addition, more rice phenology periods, like the transplanting periods and panicle development, should be extracted to analyze the phenological differences by means of ground-based spectrometers.

## 6. Conclusions

In this study, we monitored rice phenological differences under mild and severe heavy metal stress levels using remote sensing technology for exploring the potential of evaluating heavy metal stress based on rice phenology by remote sensing. We used the EVI time-series smoothed with AG function to represent the status of plant growth. Three phenological periods (the tillering period, the heading period, and the maturation period) were detected through first derivative (FD) analysis (FD_max_, FD_0_ and FD_min_) of smoothed EVI temporal profiles. Two kinds of metrics with phenological characteristics, date-intervals and time-integrated EVI, were constructed to detect rice phenological differences under varied heavy metal stress. The comparison of the two kinds of metrics indicated that the values of the metrics for presenting rice phenological differences under severe stress in the four experimental areas were smaller than the ones under mild stress. It has been proved that it is feasible to use remote sensing technology to monitor the rice phenological variations caused under different heavy metal stress, and measured the rice phenological differences with the two kinds of metrics under mild and severe heavy metal stress. This conclusion strongly supported the theory that phenology is one of the sensitive indicators of environmental change. Thus, phenology could be a useful indicator for heavy metal stress in rice plants.

## Figures and Tables

**Table 1:** The average heavy-metal concentrations in the four experimental areas.

Heavy Metals	Background Value(bi) ^1^	A		B		C		D	
		27°46′58″ N		27°58′40″ N		27°39′58″ N		27°49′15″ N	
		113°9′59″ E		113°05′56″ E		113°09′51″ E		113°02′9″ E	
		Soil (si)	Pollution Index (si/bi)	Soil (si)	Pollution Index (si/bi)	Soil (si)	Pollution Index (si/bi)	Soil (si)	Pollution Index (si/bi)
Cd	1.43	0.84	0.59	1.37	0.96	2.31	1.62	3.42	2.39
Pb	82.78	78.33	0.95	59.45	0.72	91.05	1.10	114.85	1.39
As	19.11	10.23	0.54	16.75	0.88	17.34	0.91	17.78	0.93
Pollution Level		Mild				Severe			

The unit of heavy metal concentration is mg/kg; ^1^ Background values of heavy metals were derived from the Hunan Institute of Geophysical and Geochemical Exploration, China.

**Table 2:** The statistical information on the collected soil samples: the average heavy-metal concentrations of soil, and corresponding to the standard deviation in the four experimental areas at three important rice phenological stages (initial tillering stage (mid-June), middle tillering stage (mid-July), and maturation stage (late August) marked as 1, 2, and 3, respectively).

MEAN/STD	Area A			Area B			Area C			Area D		
	1	2	3	1	2	3	1	2	3	1	2	3
**Cd**	0.678/0.009	0.881/0.007	0.952/0.004	1.26/0.013	1.38/0.005	1.47/0.009	2.22/0.01	2.35/0.003	2.36/0.008	3.11/0.15	3.31/0.009	3.84/0.005
**Pb**	68.79/0.43	88.2/0.89	78/0.77	57.45/0.78	62.9/0.54	58/0.88	83/0.65	103.15/1.59	87/0.78	111/1.08	118.55/1.23	115/0.56
**As**	7.29/0.53	11.8/0.58	11.6/0.51	16.25/0.34	17.9/0.67	16.1/0.52	16.5/0.56	18.22/0.87	17.3/0.39	15.46/0.67	19.6/0.76	18.28/0.54

**Table 3:** The standard deviation of dates corresponding to the three phenological periods(the tillering period, the heading period, the maturation period) from the four experiment areas (Area A, Area B, Area C, and Area D) were counted, respectively.

STD	Area A	Area B	Area C	Area D
Tillering	0.90	1.00	0.87	0.83
Heading	0.47	1.20	0.73	0.68
Maturation	0.84	1.45	0.74	0.81

**Table 4:** The standard deviation, maximum and minimum of L1, L2 and L3 from the four experiment areas (Area A, Area B, Area C, and Area D) were counted, respectively.

Experiment Areas	L1		L2		L3	
	STD	Max/Min	STD	Max/Min	STD	Max/Min
**Area A**	0.73	28/26	0.73	26/24	1.46	54/50
**Area B**	0.81	29/26	0.68	27/24	1.22	56/51
**Area C**	0.89	26/23	0.77	26/22	0.79	50/48
**Area D**	0.71	26/24	1.02	24/21	1.11	49/47

**Table 5:** The standard deviation of TIEVI 1, TIEVI 2, and TIEVI 3 from the four experiment areas (Area A, B, C, and D) was counted, respectively.

STD	Area A	Area B	Area C	Area D
**TIEVI1**	0.68	1.16	1.02	0.56
**TIEVI2**	0.57	1.23	0.65	0.58
**TIEVI3**	1.22	2.31	0.95	0.84
